# Effect of Spark Plasma Sintering on the Structure and Properties of Ti_1−x_Zr_x_NiSn Half-Heusler Alloys

**DOI:** 10.3390/ma7107093

**Published:** 2014-10-20

**Authors:** Ruth A. Downie, Srinivas R. Popuri, Huanpo Ning, Mike J. Reece, Jan-Willem G. Bos

**Affiliations:** 1 Institute of Chemical Sciences and Centre for Advanced Energy Storage and Recovery, School of Engineering and Physical Sciences, Heriot-Watt University, Edinburgh EH14 4AS, UK; E-Mails: rad11@hw.ac.uk (R.A.D.); s.r.popuri@hw.ac.uk (S.R.P.); 2 School of Engineering and Materials Science, Queen Mary University of London, London E1 4NS, UK; E-Mails: h.ning@qmul.ac.uk (H.N.); m.j.reece@qmul.ac.uk (M.J.R.)

**Keywords:** half-Heusler, thermoelectric, spark plasma sintering, TiNiSn, in-gap states

## Abstract

XNiSn (X = Ti, Zr and Hf) half-Heusler alloys have promising thermoelectric properties and are attracting enormous interest for use in waste heat recovery. In particular, multiphase behaviour has been linked to reduced lattice thermal conductivities, which enables improved energy conversion efficiencies. This manuscript describes the impact of spark plasma sintering (SPS) on the phase distributions and thermoelectric properties of Ti_0.5_Zr_0.5_NiSn based half-Heuslers. Rietveld analysis reveals small changes in composition, while measurement of the Seebeck coefficient and electrical resistivities reveals that all SPS treated samples are electron doped compared to the as-prepared samples. The lattice thermal conductivities fall between 4 W·m^−1^·K^−1^ at 350 K and 3 W·m^−1^·K^−1^ at 740 K. A maximum ZT = 0.7 at 740 K is observed in a sample with nominal Ti_0.5_Zr_0.5_NiSn composition.

## 1. Introduction

Half-Heusler alloys are of significant interest in the field of thermoelectrics, where they can be used in the recovery of waste heat [1,2,3]. This is largely due to naturally high Seebeck coefficients (S) and relatively large electrical conductivity values (σ), which are both key components in determining the thermoelectric efficiency (ZT) of a material. The figure of merit of a material is defined by ZT = (S^2^σ/κ)T, where the thermal conductivity (κ) is the sum of a lattice (κ_lat_) and electronic (κ_el_) component, and T is the absolute temperature. Over the past decade, a significant amount of research has been directed at improving the thermoelectric efficiencies of half-Heuslers. κ_lat_ is often the limiting factor in achieving high ZT values, thus its minimisation is of key concern. The most widely employed strategy to achieving this is isovalent substitution on the X-site in the XNiSn-based compositions. In this case, κ_lat_ is expected to decrease by introducing mass and size fluctuations leading to a disrupted phonon flow. This has been proven successful with much reduced κ-values achieved (κ = 3–4 W·m^−1^·K^−1^), leading to ZT values approaching, or in excess of, 1 [4,5,6,7,8,9,10,11]. The best performing samples generally contain either, Zr and Hf on the X site, or a mixture of Ti, Zr and Hf. The Ti_1−x_Zr_x_NiSn half-Heuslers are comparatively less well investigated and have lower ZT values but are nonetheless promising [12,13,14]. For example, we reported ZT = 0.5 for arc-melted Ti_0.5_Zr_0.5_NiSn [15] and other Ti_1−x_Zr_x_NiSn compositions have similar efficiencies [12,13,14]. Besides disorder due to alloying, the XNiSn half-Heuslers with mixed X-metals are also characterised by multiphase behaviour, *i.e.*, the presence of compositional inhomogeneities due to the poor mixing of the X-metals [16]. Recent reports suggest that this multiphase behaviour can lead to a further reduction of κ to 2–3 W·m^−1^·K^−1^ for XNiSn compositions with mixtures of Ti, Zr and Hf [10,11]. This additional reduction was not evident for samples with mixtures of only Ti and Zr which maintain κ = 3–4 W·m^−1^·K^−1^ for widely varying phase distributions [16]. A general approach to achieving much reduced κ values therefore remains elusive and the effects of synthesis and processing may be significant [17,18,19,20]. The work reported here explores the effects of spark plasma sintering (SPS) on Ti_0.5_Zr_0.5_NiSn_1−y_Sb_y_ and Ti_0.5_Zr_0.5_NiSn_0.95−y_Sb_y_ (y = 0, 0.01) samples. The 50/50 composition was chosen to maximise mass and size fluctuations on the X-site, while the Sb-doping was used to optimise the carrier concentration. The nominally Sn-deficient samples were prepared to explore the compositional stability. We have previously shown that this Sn deficiency does not persist in the final product and that Ni rich samples instead result [15]. Densification of samples is vital for determination of intrinsic thermoelectric properties, particularly the thermal conductivity, which is sensitive to porosity [21]. Such processing can, however, alter the structure and properties of a material [12,13,21,22], thus these changes must be understood in order to maximise ZT.

## 2. Results

### 2.1. Structural Properties

**Figure 1:**
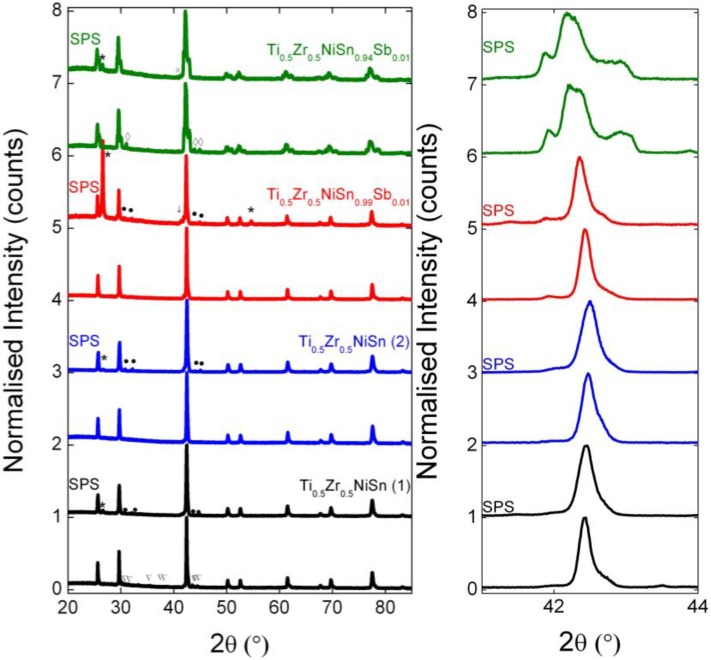
Comparison of pre- and post-SPS X-ray powder diffraction patterns for the >95% dense Ti_0.5_Zr_0.5_NiSn_1/0.95−y_Sb_y_ samples. Impurities are labelled as follows: * = graphite, ● = Sn, ∇ = Ni_3_Sn_4_, ↓ = Ti_2_Ni and ◊ = TiNi.

An overview of the prepared samples is given in Table 1. X-ray powder diffraction data collected for samples prior to SPS revealed them to be virtually pure, with minor impurities observed in Ti_0.5_Zr_0.5_NiSn(1) and Ti_0.5_Zr_0.5_NiSn_0.94_Sb_0.01_, as indicated in Figure 1. After SPS, small amounts of elemental Sn (<2 wt%) were observed in three of the four samples. Only Ti_0.5_Zr_0.5_NiSn_0.94_Sb_0.01_, which is nominally Sn-deficient, did not develop this impurity. With respect to the half-Heusler phase, broad peaks were observed in all samples, before and after SPS, as is clearly illustrated by Figure 1. This is exactly the same multiphase behaviour we observed in our previously reported Ti_1−x_Zr_x_NiSn samples Reference [16]. Analysis of the diffraction data was therefore done in an analogous manner: four to five half-Heusler phases with differing lattice parameters were used to fit each pattern. The refined lattice parameter of each of these was used to calculate the composition (x_i_) of each phase, using Vegard’s law (a = Px + Q) and these x_i_ values were combined with their respective weight fractions to produce an average composition. The microstrain (Δd/d) obtained from the profile parameters for each of the half-Heusler phases was used to calculate a further compositional variation, Δx_i_ [16]. It was therefore possible to define each sample in terms of several Ti_1−x_Zr_x_NiSn phases, each with a further associated spread in x-values. These results are collated in Table 2 for the samples pre- and post-SPS. We note that this model attributes all microstrain to compositional variations, which may not be a true representation of the sample. This is in particular the case for the SPS-treated samples where internal stresses may be present.

**Table 1:** Spark plasma sintering (SPS) conditions, pre- and post-SPS densities, and room temperature Seebeck (S) and resistivity (ρ) values for the Ti_0.5_Zr_0.5_NiSn_1/0.95−y_Sb_y_ samples.

Composition	Conditions	Density (%)		S_RT_ (μV K^−1^)		ρ_RT_ (mΩ cm)	
		Pre-SPS	Post-SPS	Pre-SPS	Post-SPS	Pre-SPS	Post-SPS
Ti_0.5_Zr_0.5_NiSn_0.95_	1000 °C/50 MPa	79	84	–	–	–	–
Ti_0.5_Zr_0.5_NiSn_0.94_Sb_0.01_	1050 °C/80 MPa	78	96	−133	−117	1.2	0.6
Ti_0.5_Zr_0.5_NiSn(1)	1050 °C/80 MPa	76	98	−286	−113	9.5	1.8
Ti_0.5_Zr_0.5_NiSn_0.99_Sb_0.01_	1050 °C/80 MPa	83	98	−141	−109	1.1	0.5
Ti_0.5_Zr_0.5_NiSn_0.98_Sb_0.02_	900 °C/50 MPa	76	83	–	–	–	–
Ti_0.5_Zr_0.5_NiSn(2)	900 °C/80 MPa	81	98	−272	−168	9.7	2.7

**Table 2:** Nominal composition, lattice parameter (a), Vegard composition (x_i_), compositional spread (Δx_i_), weight percentage (wt%), average composition (x_avg_) and goodness-of-fit (χ^2^) for pre- and post-SPS Ti_0.5_Zr_0.5_NiSn_1/0.95−y_Sb_y_ samples, as determined from X-ray powder diffraction data.

Composition	a (Å)	x_i_	Δx_i_	wt%	x_avg_	χ^2^
Ti_0.5_Zr_0.5_NiSn(1) pre-SPS	5.9915(2)	0.35(1)	0.10(1)	8.5(1)	0.52(1)	2.4
	6.0158(2)	0.48(1)	–	26.8(5)		
	6.0296(1)	0.56(1)	0.13(1)	64.6(5)		
	6.0965(8)	0.94(1)	0.13(1)	0.1(1)		
Ti_0.5_Zr_0.5_NiSn(1) post-SPS	5.9980(2)	0.38(1)	0.13(1)	7.7(2)	0.54(1)	2.2
	6.0206(1)	0.51(1)	0.13(1)	41(1)		
	6.0337(1)	0.58(1)	0.14(1)	51(1)		
	6.0972(4)	0.94(1)	0.16(1)	1.0(1)		
Ti_0.5_Zr_0.5_NiSn(2) pre-SPS	5.9995(2)	0.39(1)	0.09(1)	10.8(2)	0.52(1)	2.3
	6.0148(2)	0.48(1)	0.09(1)	19.1(5)		
	6.0278(1)	0.55(1)	0.12(1)	69.8(1)		
	6.100(1)	0.96(1)	0.04(1)	0.4(2)		
Ti_0.5_Zr_0.5_NiSn(2) post-SPS	5.9990(2)	0.39(1)	0.12(1)	8.9(2)	0.55(1)	3.5
	6.0170(1)	0.49(1)	0.07(1)	16.3(3)		
	6.0324(1)	0.58(1)	0.18(1)	73.5(3)		
	6.0972(4)	0.94(1)	0.10(1)	1.2(1)		
Ti_0.5_Zr_0.5_NiSn_0.99_Sb_0.01_ pre-SPS	5.9929(2)	0.35(1)	0.23(1)	12.9(2)	0.54(1)	2.1
	6.0211(1)	0.51(1)	0.09(1)	28.2(5)		
	6.0329(1)	0.58(1)	0.09(1)	57.3(5)		
	6.0995(2)	0.95(1)	0.05(1)	1.6(1)		
Ti_0.5_Zr_0.5_NiSn_0.99_Sb_0.01_ post-SPS	5.9947(3)	0.36(1)	0.17(1)	7.9(2)	0.57(1)	1.6
	6.0230(3)	0.52(1)	0.17(1)	31(1)		
	6.0358(1)	0.59(1)	0.13(1)	56(1)		
	6.0953(6)	0.93(1)	0.23(1)	5.2(2)		
Ti_0.5_Zr_0.5_NiSn_0.94_Sb_0.01_ pre-SPS	5.9573(2)	0.15(1)	0.26(1)	14.9(3)	0.56(1)	2.8
	5.9834(5)	0.30(1)	0.25(1)	6.8(2)		
	6.0346(2)	0.59(1)	0.21(1)	41.6(6)		
	6.0600(1)	0.73(1)	0.20(1)	33.3(7)		
	6.0991(1)	0.95(1)	0.03(1)	3.7(1)		
Ti_0.5_Zr_0.5_NiSn_0.94_Sb_0.01_ post-SPS	5.9624(3)	0.18(1)	0.29(1)	15.2(3)	0.58(1)	2.0
	5.9947(4)	0.36(1)	0.18(1)	5.3(2)		
	6.0351(2)	0.59(1)	0.26(1)	37.8(9)		
	6.0588(2)	0.72(1)	0.24(1)	36.4(1)		
	6.0982(2)	0.95(1)	0.06(1)	5.4(2)		

Space group: *F-43m*, Ti/Zr on site *4a* (0,0,0), Ni on *4c* (0.25, 0.25, 0.25) and Sn on *4b* (0.5, 0.5, 0.5).

Comparison of the lattice parameters reveals small increases after SPS (Table 2), suggesting that the samples become richer in Zr. For example, the Ti_0.5_Zr_0.5_NiSn(1) sample has a pre-SPS experimental composition of Ti_0.48(1)_Zr_0.52(1)_NiSn which changes to Ti_0.46(1)_Zr_0.54(1)_NiSn post-SPS. Similarly, the nominal Ti_0.5_Zr_0.5_NiSn_0.99_Sb_0.01_ sample changes from a pre-SPS composition of Ti_0.46(1)_Zr_0.54(1)_NiSn_0.99_Sb_0.01_ to Ti_0.43(1)_Zr_0.57(1)_NiSn_0.99_Sb_0.01_. The increases in lattice parameter are accompanied by an increase in the overall peak width of the half-Heusler reflections. This may be observed in Figure 1 and is also apparent from the Δx values (Table 2), which are generally larger post-SPS. As already mentioned, it is possible that this broadening is attributable to stresses in the sample and do not reflect further changes in the sample composition.

**Figure 2:**
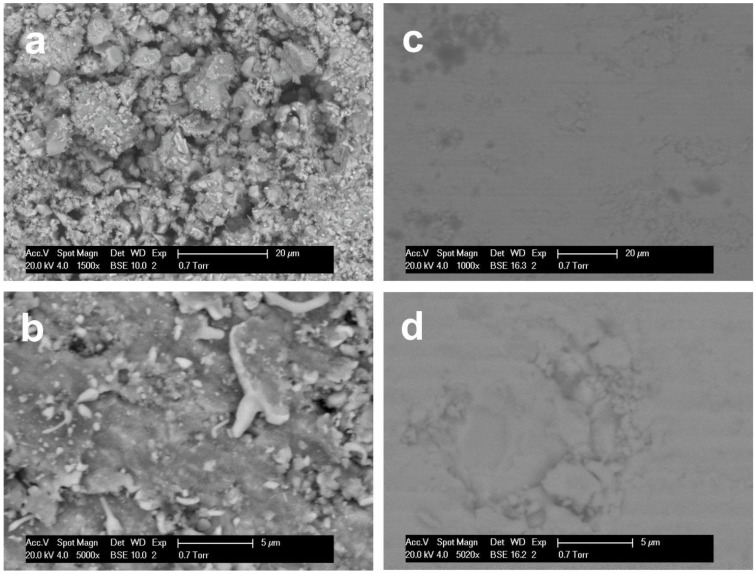
SEM images of Ti_0.5_Zr_0.5_NiSn(1) pre-SPS (**a**,**b**) and post SPS (**c**,**d**).

SEM images collected for Ti_0.5_Zr_0.5_NiSn(1) before and after SPS are presented in Figure 2. Prior to SPS, the sample was characterised by numerous highly textured areas, as illustrated in Figure 2a, where the grains are not well-sintered. Even in apparently smoother areas, close inspection reveals a relatively rough, porous surface (Figure 2b). Comparison with images taken post-SPS shows this to have a much smoother surface with very few features visible (Figure 2c,d). This is indicative of better sintered grains and a loss of porosity, as may be expected from the increase in density (Table 1).

### 2.2. Thermoelectric Properties

**Figure 3:**
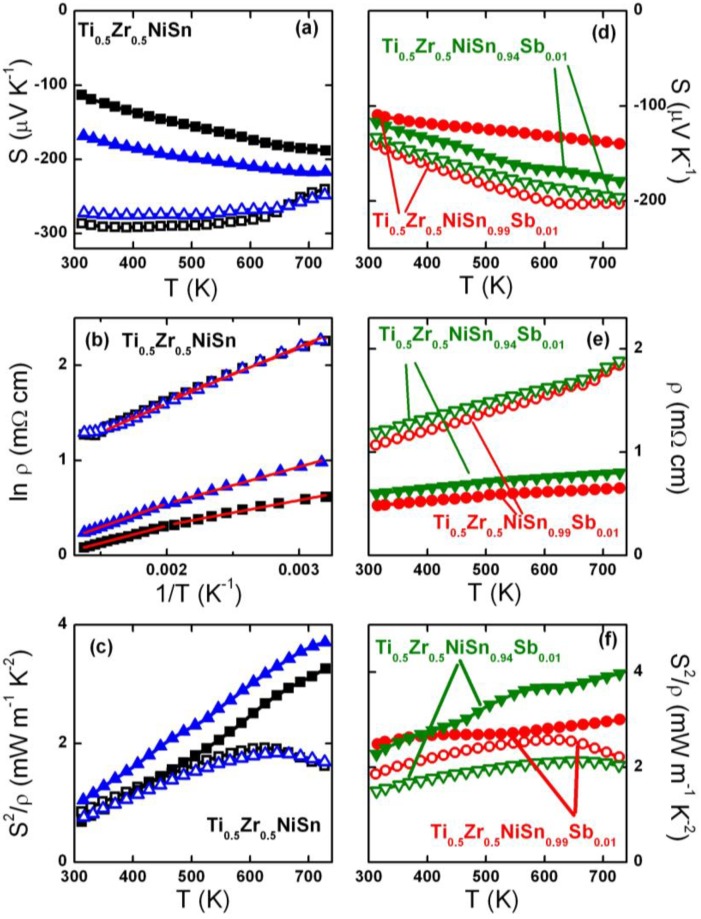
Temperature dependence of Seebeck coefficient (S), resistivity (ρ) and power factor (S^2^/ρ) for the Ti_0.5_Zr_0.5_NiSn (**a**–**c**) and the Ti_0.5_Zr_0.5_NiSn_1/0.95−y_Sb_y_ (**d**–**f**) samples. Open symbols represent the pre-SPS samples and the filled symbols are for the post-SPS samples. Red lines in (**b**) correspond to Arrhenius fits to the data, as detailed in Table 3.

A comparison of S(T) and ρ(T) for the Ti_0.5_Zr_0.5_NiSn_1/0.95−y_Sb_y_ samples pre- and post-SPS densification is shown in Figure 3. As expected for samples with reduced porosity a strong decrease in ρ(T) is apparent in each case. The ρ values of the two Ti_0.5_Zr_0.5_NiSn samples are reduced by a factor of 5 at room temperature, and a factor of 4 at 730 K. The Sb-doped samples show an approximately twofold reduction in resistivity over the whole temperature range. A plot of lnρ(T) versus inverse temperature shows two linear regions for the Ti_0.5_Zr_0.5_NiSn samples, both pre- and post-SPS. A subtle transition occurs near 500 K. The slopes were used to obtain an estimate of the activation energy (E_a_ = E_g_/2; where E_g_ is the band gap) for electron transport, which is ~0.05 eV (E_g_ = 0.1 eV) for the pre-SPS samples, and between 0.025 eV and 0.045 eV for the post-SPS samples. These activation energies are smaller than expected from the band gap values reported from high-temperature (>700 K) ρ(T) data, which yield E_g_ = 0.2–0.3 eV [23,24,25]. The ρ(T) for the Sb doped samples could not be fitted using either a thermally activated or variable range hopping model for electronic conduction.

The S(T) show considerable changes in magnitude for all of the samples after SPS-treatment. Since S does not depend on porosity changes in its value reflect changes in electronic transport upon densification. In particular, the observed reductions are consistent with n-type doping. The change is most pronounced for the two Ti_0.5_Zr_0.5_NiSn samples, where a clear change in temperature dependence is observed. Prior to SPS, S is large at RT and decreases upon heating, while after SPS, S is small at RT and increases upon heating. The impact on the already electron doped (Sb doped) samples is smaller. Nonetheless substantial reductions consistent with carrier doping are observed. The resulting power factors (S^2^/ρ) reach a maximum of 3.75 mW·m^−1^·K^−2^ for Ti_0.5_Zr_0.5_NiSn(2), and of 4 mW·m^−1^·K^−2^ for Ti_0.5_Zr_0.5_NiSn_0.94_Sb_0.01_ at 740 K (Figure 3c,f).

**Table 3:** Activation energy (E_a_) and exponential pre-factor (ρ_0_) for the Ti_0.5_Zr_0.5_NiSn samples, as determined by an Arrhenius-fit to the resistivity data (see Figure 3).

Sample		T range	Ea (eV)	ρ_0_ (mΩ cm)
1	Pre-SPS	300–500 K	0.046(2)	1.8(1)
		500–650 K	0.055(2)	1.48(7)
	Post-SPS	300–500 K	0.023(1)	0.81(3)
		500–730 K	0.032(1)	0.66(1)
2	Pre-SPS	300–500 K	0.051(2)	1.58(5)
		500–650 K	0.054(5)	1.48(7)
	Post-SPS	300–500 K	0.034(2)	0.79(2)
		500–730 K	0.044(1)	0.64(1)

**Figure 4:**
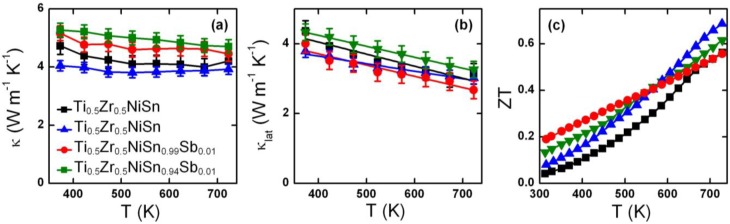
Temperature dependence of (**a**) thermal conductivity (κ); (**b**) lattice thermal conductivity (κ_lat_) and (**c**) ZT for the Ti_0.5_Zr_0.5_NiSn_1/0.95−y_Sb_y_ samples.

The temperature dependence of κ and κ_lat_ and the figure of merit, ZT, for the dense samples, are given in Figure 4. The lattice contribution was calculated using κ−κ_el_ = κ−LT/ρ where a Lorenz factor, L = 1.6 × 10^−8^ W·Ω·K^−2^, calculated by Muta *et al.* [26] was used. The total thermal conductivity is almost temperature independent in the 350–740 K interval. The Ti_0.5_Zr_0.5_NiSn samples have κ = 4–5 W·m^−1^·K^−1^ over the whole temperature range, consistent with the values measured for our previously reported arc-melted Ti_0.5_Zr_0.5_NiSn samples [15], and samples prepared by solid state reactions [16]. The electron doped Ti_0.5_Zr_0.5_NiSn_0.99_Sb_0.01_ and Ti_0.5_Zr_0.5_NiSn_0.94_Sb_0.01_ compositions have higher values (5–5.5 W·m^−1^·K^−1^), consistent with a larger electronic contribution due to the increased amount of charge carriers. The lattice thermal conductivities are very similar for the four measured samples and decrease linearly from ~4 W·m^−1^·K^−1^ at 350 K to ~3 W·m^−1^·K^−1^ at 740 K. The temperature dependence of ZT is steeper for the non electron doped samples with a maximum ZT = 0.7 at 740 K for Ti_0.5_Zr_0.5_NiSn, while the Sb-doped samples achieve ZT ≈ 0.6 at the same temperature.

## 3. Discussion

SPS was used to prepare >95% dense Ti_0.5_Zr_0.5_NiSn_1/0.95−y_Sb_y_ samples. The multiphase behaviour that occurs in these samples is maintained post-SPS but small changes in composition and some additional peak broadening are evident. The changes in composition can be inferred from the increases in lattice parameters, which within our model leads to an apparent increase in Zr content. However, this may also reflect another compositional change that results in an increase in lattice parameter. For example, small amounts of elemental Sn (<2 wt%) are visible in the X-ray data after densification, and it may be that Sn is extruded, leaving residual Ti and Ni atoms to occupy the vacant tetrahedral site in the half-Heusler structure, leading to increased lattice parameters [15]. The observed peak broadening is most likely linked to stresses arising from the SPS treatment but could also be linked to the observed changes in composition. The literature is not clear regarding the impact of SPS on the peak shapes as a narrowing was observed for Ti_0.3_Zr_0.7_NiSn [12] but broadening and even peak splitting were observed for (Zr_0.6_Hf_0.4_)_0.7_Ti_0.3_NiSn [21]. One notable difference is that solid state reactions were used to prepare the pre-SPS samples in this study, while arc-melting was utilised in the literature examples.

It is clear from the S(T) and ρ(T) data that SPS densification results in changes to the electronic transport. The observed reductions in S(T) and ρ(T) are consistent with electron doping, which is in keeping with the subtle changes in composition observed in our diffraction analysis. Even without changes to the half-Heusler composition the presence of metallic impurities (Sn) may result in electron doping. The combined reduction of porosity and electron doping results in increases in S^2^/ρ for all samples after SPS. The largest S^2^/ρ values are near 4 mW·m^−1^·K^−2^ at 740 K. In principle, a constant S(T) (*i.e.* no electron doping) and just a reduction in ρ(T) (solely due to a reduced porosity) would have been preferred in order to undertake systematic studies of the loss of porosity. Our data suggests that the ultimate performance of these samples has been compromised. For example, maintaining S_RT_ = −286 μV K^−1^ and reducing ρ by 50% to 5 mΩ cm for Ti_0.5_Zr_0.5_NiSn(1) yields S^2^/ρ = 1.6 mW·m^−1^·K^−2^ at RT compared to the measured value of 0.7 mW·m^−1^·K^−2^. In fact, for this particular sample, the pre-SPS power factor at RT is 0.8 mW·m^−1^·K^−2^, which is larger than the value obtained post-SPS, although at elevated temperatures clear improvements are observed.

The activation energies for carrier transport for the Ti_0.5_Zr_0.5_NiSn samples in Table 3 provide a useful insight into the electronic structure of these materials. The fitted activation energies are on the order of 0.05 eV (corresponding to E_g_ = 0.1 eV) which is considerably smaller than the E_g_ = 0.2–0.3 eV reported from high-temperature ρ(T) data. These small values are not unprecedented, for example, similar values (E_a_ ~ 0.05 eV) have been reported for Zr_1−x_Hf_x_NiSn samples from fits between 100 K and 330 K [27]. It therefore appears that at low temperatures the resistivity measurements probe a smaller effective band gap. One possible explanation is the presence of in-gap states which have been widely reported in the literature [28,29]. Computational studies have shown that these can arise from a partial disordering of the metal atoms over the X, Ni and Sn positions but also from the partial occupancy of the vacant tetrahedral site in the half-Heusler structure [30]. Compositions with intentional Ni excess have recently started to attract interest [15,31]. The reduction in the post-SPS activation energies is consistent with the presence of additional in-gap states which reduce the effective band gap for carrier excitation (the average ΔE_a_ = −0.02 eV; −40%). Our diffraction analysis suggests that interstitial Ni and/or Ti are introduced and that Sn is extruded. This should result in an increased amount of in-gap states and a smaller activation energy, consistent with the experimental data [32]. Furthermore, with increasing temperature the contribution from the in-gap states diminishes as more and more carriers are excited directly across the “intrinsic” band gap. Experimentally, the fitted activation energies indeed show a modest increase and are on average 0.008 eV (+15%) larger in the 500–700 K regime. For measurements above 700 K we expect to recover band gap values in line with the literature values of 0.2–0.3 eV. If this interpretation is correct, it suggests that in-gap states (small amounts of interstitial Ni and/or Ti) are almost always present, and that the amount is sensitively dependent on sample processing, which may help explain the widely varying reported properties for nominally similar compositions [1,3].

The availability of dense samples allowed an accurate measurement of the thermal conductivity. The total (4–5 W·m^−1^·K^−1^) and lattice (3–4 W·m^−1^·K^−1^) thermal conductivities are typical of the Ti_1−x_Zr_x_NiSn series [15,16], and remain somewhat higher than the 2–3 W·m^−1^·K^−1^ reported for samples with mixtures of Ti, Zr and Hf [5,10,11]. This results in respectable ZT values up to 0.7 at 740 K for the Ti_0.5_Zr_0.5_NiSn(2) sample. These values are moderately large, with Ti_1−x_Zr_x_NiSn samples usually achieving ZT = 0.3–0.5 at around 800 K [13,14,33].

## 4. Materials and Methods

Ti_0.5_Zr_0.5_NiSn_1−y_Sb_y_ (y = 0, 0.01, 0.02) and Ti_0.5_Zr_0.5_NiSn_0.95−y_Sb_y_ (y = 0, 0.01) samples were prepared on a 5 g scale by standard solid state reactions. Stoichiometric quantities of the elemental starting materials (>99.9% purity, obtained from Alfa Aesar) were ground in an agate mortar and pestle. The mixtures were then cold-pressed into pellets, vacuum sealed in carbon coated quartz tubes and annealed at 900 °C for 24 hours. Samples were subsequently re-ground, cold-pressed and resealed in carbon coated quartz tubes. They were then annealed at 900 °C for a further 2 weeks. An additional Ti_0.5_Zr_0.5_NiSn sample (referred to as Ti_0.5_Zr_0.5_NiSn(2) herein) was prepared in a similar manner but the pellets were wrapped in Ta foil prior to sealing within the quartz tube. After the conventional sintering steps, a portion was removed for X-ray powder diffraction and a bar was cut for electronic property measurements. These samples are referred to herein as pre-SPS. The remainder of each sample was then ground to a fine powder and sintered by Spark Plasma Sintering (HPD-25/1, FCT, Systeme GmbH, Frankenblick, Germany). All samples were pressed for 3 min with a ramp rate of 100 °C/min. Several temperatures and pressures were used in order to determine optimum SPS conditions. These samples are denoted “post-SPS”. X-ray diffraction analysis revealed that samples sintered at 1000 °C and 1050 °C contained a strong impurity peak associated with graphite, as may be observed in Figure 1. Optimum conditions were therefore determined to be 900 °C and 80 MPa. The structure and properties of samples that attained >95% density were subsequently analysed.

Laboratory X-ray powder diffraction patterns were collected on a Bruker D8 Advance diffractometer (Billerica, MA, USA) with monochromated Cu K_α1_ radiation. Datasets of 8 h were used for Rietveld analysis. Rietveld fits were performed using the GSAS and EXPGUI suite of programs [34,35]. Scanning electron microscopy was performed using a FEI Quanta 650 FEG ESEM (Eindhoven, The Netherlands) operated in low vacuum at 60 Pa. A voltage of 20 KV was used, with a working distance of 10 mm. The temperature dependence of the Seebeck coefficient and electrical resistivity were measured between 35 °C and 500 °C using a Linseis LSR-3 (Selb, Germany) high temperature Seebeck and resistance probe. The thermal conductivity was measured between 50 °C and 500 °C using an Anter Flashline 3000 (now TA instruments, New Castle, DE, USA) flash diffusion instrument using a Pyroceram reference sample. A porosity correction: κ/κ_dense_ = 1 − (4/3)ϕ, where ϕ = (100% − %density)/100, was applied. 

## 5. Conclusions

To conclude, SPS densification of previously well characterised Ti_0.5_Zr_0.5_NiSn_1/0.95−y_Sb_y_ compositions has led to changes in composition and electron doping. This may have reduced the maximum attainable power factors, and thereby the energy conversion efficiency. A largest ZT = 0.7 at 740 K was observed for a Ti_0.5_Zr_0.5_NiSn sample.
